# Saving Mothers, Giving Life: Don’t Neglect the Health Systems Element

**DOI:** 10.9745/GHSP-D-19-00178

**Published:** 2019-12-23

**Authors:** Krishna Hort, Louise Simpson

**Affiliations:** a Nossal Institute for Global Health, University of Melbourne, Melbourne, Australia.; b Australia Indonesian Partnership for Maternal and Neonatal Health, Kupang, Indonesia.

See related articles in the SMGL supplement.

## INTRODUCTION

We congratulate the authors of the articles in the GHSP supplement on the Saving Mothers, Giving Life (SMGL) project in Uganda and Zambia. The significant reduction in maternal deaths arising from the project is heartening, and we are pleased to see this comprehensive description of the project, its interventions and outcomes, and a range of studies evaluating its impact, all published in full.

In this letter, we would like to focus on an aspect of the project that we feel did not receive adequate attention in the supplement, namely, its role as a health systems strengthening (HSS) initiative. To do so, we draw on the literature describing the characteristics of HSS initiatives and seek to highlight the HSS elements of the SMGL program based on the articles in the supplement. Given the relatively sparse literature on HSS characteristics, we also draw on our own experience of HSS in relation to maternal health programs through the Australia-Indonesia Partnership for Maternal and Neonatal Health (AIPMNH) in eastern Indonesia, over the period 2009 to 2015 (unpublished).

## HEALTH SYSTEMS STRENGTHENING

The SMGL initiative was clearly conceived as an HSS initiative, using a “systems approach, focused at the health district level”^1^ and addressing 5 elements of the health system in an integrated manner. This systems approach was designed to “create a highly visible, bold initiative that would galvanize global action and financial support”^2^ and demonstrate that such an initiative “could achieve impressive results in a short time.”^1^

However, literature on HSS emphasizes that it goes beyond simply addressing health system components. HSS involves^3^:

*investments in inputs in an integrated and systemic way, but also reforming the architecture that determines how different parts of the health system operate and interact to meet priority health needs through people-centered integrated services.*

An HSS approach also takes the complex and adaptive nature of health systems into consideration,^4^ which has given rise to the view that HSS is also^5^:

*a complex, iterative, and learning process wherein the interactions between actors, structures, services, and subsystems are optimized over time while striving for health systems goals.*

Based on this perspective, evaluations of HSS should include the process of implementation to understand how the HSS intervention interacts with and adapts to the operating environment.^4^ However, the implementation process is not always well captured in evaluations of HSS interventions, as noted by Adam et al.^4^ in their review of studies of HSS.

Unfortunately, the collection of articles on the SMGL initiative has also somewhat neglected the implementation process element. The focus of reporting and evaluation appears to have been on the specific interventions and their links to results and outcomes. As noted by one of the SMGL authors^6^:

*Although extensive monitoring and evaluation activities were implemented for SMGL, these methods focused heavily on measuring effects on health outcomes and much less on process documentation of various programmatic approaches.*

The minimal focus on process documentation occurred despite the fact that^7^:

*the majority of the interventions supported by SMGL were not “new” to the host country; rather, they were existing interventions that were refined, strengthened, and, in most cases, taken to greater scale of implementation through partnership*.

Consequently, the implementation process seems to have in fact been an important element of the SMGL initiative. With this in mind, we reviewed the articles to try to identify where and how the project adapted to different contexts, responded to contextual changes, and evolved during the implementation process.

## LEVELS OF IMPLEMENTATION

To identify the implementation elements, we use the framework proposed by Samuels et al.^8^ and the approach taken by Cleary et al.^9^ to describe an HSS project in Mozambique. Samuels et al.^8^ referred to 3 levels of implementation—macro-level governance, meso-level partnerships, and micro-level local ownership.

### Macro Level

At a macro level, Samuels et al.^8^ identified the following factors as being key to implementation: effective governance, coherent evidence-based policies, and partnerships between donors and national-level actors that encourage the latter to take control and enable transition from donor funds to national funding streams. Cleary et al.^9^ also identified the importance of “relational trust building” with partners in terms of interpersonal and institutional trust as contributing to implementation success.

The SMGL articles refer to implementation at a macro level in managing the multiple partnerships involved and in gaining national government support and commitment. They report some success in building high levels of ownership among district health leaders, as well as “increased [Ministry of Health] commitment and heightened social awareness” at the national level with regard to the prevention of maternal and newborn deaths.^2^ However, the papers contain little information on how these relationships were built.

One challenge to these relationships appears to have been the funding “gap” that occurred between funding for the initial year and a decision on whether to continue funding for a longer period.^2^ Erratic funding in subsequent years was also mentioned. Thus, project implementers needed to manage the “frustration” among government and implementing partners generated by these funding requirements. Learning how this management was accomplished would be interesting.

SMGL used a funding mechanism designed to encourage increasing national government contribution through progressive annual reductions in funds provided.^2^ However, the impacts of this process on relationships were not discussed, although the failure to gain increased government funding mentioned by Healey et al.^7^ suggests that such impacts may well have contributed a further implementation challenge.

Our experience in Indonesia also demonstrates the critical importance of developing partnership with key government managers; building partner government capacity in leadership and governance, which requires long-term commitment (5 years plus in our case); and ensuring close collaboration in decision making.

### Meso Level

Samuels et al.^8^ described the meso level as the level at which:

*policies become specific interventions shaped by organizational structures and procedures, and partnerships among different organizations.*

Such interventions occur primarily at a subnational level and are closely related to the capacity and context of district administration, for example, the extent of decentralization.

SMGL implementation at a district level relied on multiple partners, including teams of Ugandan and Zambian government medical and local civic leaders as well as “equally dedicated and talented U.S. government teams.”^1^ This approach was reported to have enabled^1^:

*considerable problem solving, resource gathering, and resilience in the face of unexpected administrative and logistical challenges.*

In addition, the approach was reported to have contributed to addressing both supply- and demand-side barriers that accelerated change.^2^

The SMGL articles describe some implementation challenges associated with this approach. For example, in Zambia, the tools and systems for facility data collection were developed separately by individual partners and were not harmonized across districts; consequently, some indicators could not be aggregated at baseline.^10^ In addition, the management of multiple partnerships created a heavy administrative burden, and the SMGL authors recommended a smaller partnership in the future.^2^

The meso level is also where implementation might encounter contextual changes and need to adapt to changing circumstances. The SMGL authors referred to changes in the district structure in both countries and to adaptations during implementation to address issues of ambulance sustainability, transport voucher demand, and scope, such as the expansion to include postpartum and neonatal care.^2^ However, the articles contain no reference to the extent or implications of decentralization, which operates in both countries and represents an important contextual factor.

The articles address the need to develop capacity at the district level in “planning, execution, and evaluation,”^2^ and to the use of national intermediaries to support implementation. SMGL district coordinators—often retired midwives—were hired to harmonize all SMGL activities in their district with district health officers and district health management teams, and to serve as a link with implementing partners.^2^

Our experience in the AIPMNH was similar. Although district governments in Indonesia operated in a highly decentralized environment, district capacity in planning and execution was low. We found that the provision of flexible funding from the project enabled district governments to introduce new approaches and activities that could not be funded through the complex government planning and budget process. However, planners needed training and support in the planning and execution process to ensure proposed activities were appropriate and effectively implemented.

### Micro Level

At a micro level, the SMGL articles describe extensive use of community extension workers to engage with communities and to provide a bridge between health services and pregnant women and their families. The workers included village health team volunteers in Uganda and Safe Motherhood Action Groups in Zambia. In both countries, the workers advocated for birth preparedness, promoted healthy practices, and encouraged antenatal care visits, facility deliveries, and postpartum care.^6^

We also used this strategy in Indonesia, building on and strengthening existing village and community institutions, such as the health post (posyandu) concept to support safe pregnancy and delivery, and engaging with church groups, which wield considerable influence in this largely Christian area.

One of the key challenges for these community-level activities is ensuring sustainability, and we were heartened to see that Healey et al.^7^ commented that the formalization and institutionalization of the community volunteer groups was one of the most significant signs of sustainability.

### Learning and Adaptation

The SMGL papers also imply a learning and adaptive process during implementation, with Conlon et al. referring to the development of a “think tank” atmosphere toward the latter stages of the project.^2^ This process is of considerable interest to us, because we also noted how the AIPMNH program evolved from a focus on implementation toward support for the development and conduct of studies, interventions and evaluations of innovative practices, and the exchange of this information across districts and nationally.

## DISCUSSION

The implementation experience of SMGL, although not well documented, supports many of the aspects identified by Cleary et al.^9^ regarding the HSS project in Mozambique. They noted the need to develop ownership, build trust, adapt to contextual change, and have long-term adaptive support.

However, it is worth noting that Cleary et al.^9^ stressed the need for long-term funding commitment and for flexibility in scheduling, for example, through lengthy start-up phases.^9^ This area appears to be one in which the SMGL approach of initial “conditional” funding for the start-up year, followed by a 6-month “chaotic” period waiting for a decision on implementation, was not in line with best practice in HSS and resulted in a recommendation that^2^:

*any future systems approach should commit to a minimum of 5 years support from the outset.*

Finally, we note that, although choosing the right strategies and interventions is important in a systems approach, the implementation process and managing implementation can be just as important. In a journal devoted to science and practice, let us not neglect the “practice” element.

This point was well made by Cleary et al.^9^:

*However, implementation practice in HSS is rarely reported in close detail, and these “obvious” issues are rarely intentionally managed, reported, or measured. This case study shows how important implementation practice can be as it underpins HSS intervention activities and their success—and suggests that it may need to be taken more seriously into account by funders, intervention designers, implementers, and researchers—as a key element of intervention design, management, and evaluation.*

## Notes

**First Published Online:** October 17, 2019
